# Unexpected Phonon Behaviour in BiFe_x_Cr_1−x_O_3_, a Material System Different from Its BiFeO_3_ and BiCrO_3_ Parents

**DOI:** 10.3390/nano12091607

**Published:** 2022-05-09

**Authors:** Cameliu Himcinschi, Felix Drechsler, David Sebastian Walch, Akash Bhatnagar, Alexei A. Belik, Jens Kortus

**Affiliations:** 1 Institute of Theoretical Physics, TU Bergakademie Freiberg, D-09596 Freiberg, Germany; felix.drechsler@student.tu-freiberg.de (F.D.); jens.kortus@physik.tu-freiberg.de (J.K.); 2 Zentrum für Innovationskompetenz SiLi-nano, Martin-Luther-Universität Halle-Wittenberg, D-06120 Halle (Saale), Germany; david.knoche@physik.uni-halle.de (D.S.W.); akash.bhatnagar@physik.uni-halle.de (A.B.); 3 Institut für Physik, Martin-Luther-Universität Halle-Wittenberg, D-06120 Halle (Saale), Germany; 4 International Center for Materials Nanoarchitectonics (WPI-MANA), National Institute for Materials Science (NIMS), Namiki 1-1, Ibaraki, Tsukuba 305-0044, Japan; alexei.belik@nims.go.jp

**Keywords:** Raman spectroscopy, thin films, spectroscopic ellipsometry, charge transfer, phase transition, multiferroics, BiFe_x_Cr_1−x_O_3_

## Abstract

The dielectric function and the bandgap of BiFe_0.5_Cr_0.5_O_3_ thin films were determined from spectroscopic ellipsometry and compared with that of the parent compounds BiFeO_3_ and BiCrO_3_. The bandgap value of BiFe_0.5_Cr_0.5_O_3_ is lower than that of BiFeO_3_ and BiCrO_3_, due to an optical transition at ~2.27 eV attributed to a charge transfer excitation between the Cr and Fe ions. This optical transition enables new phonon modes which have been investigated using Raman spectroscopy by employing multi-wavelengths excitation. The appearance of a new Raman mode at ~670 cm^−1^ with a strong intensity dependence on the excitation line and its higher order scattering activation was found for both BiFe_0.5_Cr_0.5_O_3_ thin films and BiFe_x_Cr_1−x_O_3_ polycrystalline bulk samples. Furthermore, Raman spectroscopy was also used to investigate temperature induced structural phase transitions in BiFe_0.3_Cr_0.7_O_3_.

## 1. Introduction

Bi_2_FeCrO_6_ was first theoretically proposed as a bismuth-based multiferroic material having large spontaneous magnetisation and polarisation by means of first principle studies by Baettig et al. [1,2]. Following this theoretical prediction, Bi_2_FeCrO_6_ epitaxial films have also been synthesised and its multiferroic character demonstrated [3,4]. Consequently, a lot of attention was dedicated to the characterisation of structural, ferroelectric and multiferroic properties of the material, and to the optimisation of the growth parameters for thin films [5,6,7,8]. Promising photovoltaic properties, the low bandgap and the possibility to tune it by the growth conditions, cationic ordering, and the domain size have attracted a lot of attention in recent years [9,10,11,12,13]. Various heterostructures or BiCrO_3_/BiFeO_3_ superlattices with different thicknesses and repetitions have been proposed for designing efficient ferroelectric photovoltaic devices [14,15,16].

Raman spectroscopy is an established method to detect subtle structural changes induced by strain or temperature and it has been already very successfully applied to the study of the lattice dynamics in the class of oxides with perovskite structures. However, in the case of Bi_2_FeCrO_6_ there are very few Raman or infrared spectroscopic studies reported [17,18,19].

In the present paper Raman spectroscopy was used in combination with spectroscopic ellipsometry for the characterisation of BiFe_0.5_Cr_0.5_O_3_ thin films and polycrystalline BiFe_x_Cr_1−x_O_3_ materials. It was thus possible to provide the evidence of light induced phonon modes and to follow the temperature induced phase transitions by Raman spectroscopy in these material systems. It should be mentioned that Bi_2_FeCrO_6_ is commonly used in literature for double perovskite structure with alternating Fe and Cr in the unit cell [14]. BiFe_0.5_Cr_0.5_O_3_, although an equivalent representation of the same stoichiometry, allows for some deviation from the ideal 1:1 ordering expected from a typical Bi_2_FeCrO_6_ unit cell. In this paper, for the sake of simplicity and consistency, BiFe_0.5_Cr_0.5_O_3_ (BFCO) is used for the stoichiometry of the films which also allows us to address other investigated stoichiometries for BiFe_x_Cr_1−x_O_3_ bulk samples.

## 2. Materials and Methods

Epitaxial BiFe_0.5_Cr_0.5_O_3_ films with thickness of 120–200 nm were grown on SrTiO_3_ (001)_c_ substrates using pulsed laser deposition (SURFACE systems + technology PLD-Workstation (Hueckelhoven, Germany), Coherent KrF excimer laser (Santa Clara, CA, USA)). A stoichiometric ceramic BiFe_0.5_Cr_0.5_O_3_ target was placed 60 mm below the substrate. During the deposition the substrate was kept at a temperature of 700 °C and exposed to 0.01 mbar oxygen partial pressure inside the chamber. The laser pulses were set to a pulse energy of 136 mJ with a repetition rate of 2 Hz. After finishing the deposition, the film was cooled at a high oxygen partial pressure (>200 mbar) with a rate of 5 °C/min. The crystallinity and phase purity of the BFCO films were confirmed by X-ray analysis (see Figure S1 in Supplementary Materials). BiFe*_x_*Cr_1−*x*_O_3_ (*x* = 0.3, 0.4, 0.6, 0.7) bulk polycrystalline samples were prepared from stoichiometric mixtures of Bi_2_O_3_ (99.9999%), Fe_2_O_3_ (99.999%), and Cr_2_O_3_ (99.9%) (Rare Metallic Co. Ltd., Tokyo, Japan) at high-pressure, high-temperature conditions of about 6 GPa. The sample with *x* = 0.7 was annealed at about 1550 K in an Au capsule for 90 min. The samples with *x* = 0.3, 0.4, 0.6 were annealed at about 1700 K in Pt capsules for 60 min. After annealing, quenching was performed by turning off heating current, and then pressure was slowly released.

The ellipsometric measurements were performed at room temperature at four angles of incidence (55°, 60°, 65° and 70°) using a M2000 ellipsometer from J.A. Woollam Company (Lincoln, NE, USA).

The micro-Raman measurements were performed with a LabRam Horiba Jobin Yvon spectrometer (Villeneuve d’Ascq, France) using 325, 442, 532, and 633 nm laser lines for excitation. A 50× magnification objective (N.A. 0.55) was used to focus and collect the scattered light, while the laser power was set low enough (below 1.5 mW for all lasers) in order to avoid damage of the samples surface and any influence of laser induced heating on the Raman spectra. All spectra were recorded in backscattering geometry. Temperature-dependent Raman measurements from 90 K up to 700 K were carried out using a LinkamTHMS-600 cooling stage (Salfords Redhill, UK) and a Linkam TS 1200 heating chamber (Salfords Redhill, UK) placed under the Raman microscope.

## 3. Results and Discussions

### 3.1. Ellipsometry Dielectric Function

First, a bare SrTiO_3_ substrate was measured using ellipsometry, its optical response being modelled using a sum of Gaussian oscillators which ensures the Kramer–Kronig consistency of the real and imaginary parts of dielectric function. The determined dielectric function of the SrTiO_3_ substrate was further used for the evaluation of the film/substrate system. In a second step, the absorption free energy range (below 1.75 eV) was used in order to estimate the thickness of the BFCO films using a four-layer model (substrate/film/roughness layer/ambient). The procedure used here was also applied previously for the determination of the optical constants of BiFeO_3_ films [20]. The equations relating the ellipsometric parameters to the dielectric function can be found in [21]. The optical response of the film was modelled by a Cauchy dispersion relation ($n=A_{n}+B_{n}/\lambda^{2})$ (describing the refractive index in this spectral range. In a third step, using the thickness and the roughness obtained in the second step as an initial guess, the ellipsometric data were evaluated in the energy range up to 6 eV describing the line shape of the imaginary part of the dielectric function of the film as a sum of Gaussian oscillators, while its real part is generated according to the Kramers–Kronig relation.

Using the method mentioned above a thinner (~120 nm) and a thicker (~200 nm) film with an optical roughness of ~4 nm, were evaluated using a multi-sample analysis method in order to avoid correlations between the fit parameters of the dielectric function and the thickness. Evaluating the films separately delivered nearly the same results, with an error in the determination of the thickness and roughness of ± 1 nm, which indicate that both films have the same optical properties. The thickness values obtained from ellipsometry were in good agreement with the expected nominal thickness from the PLD growth.

**Figure 1:**
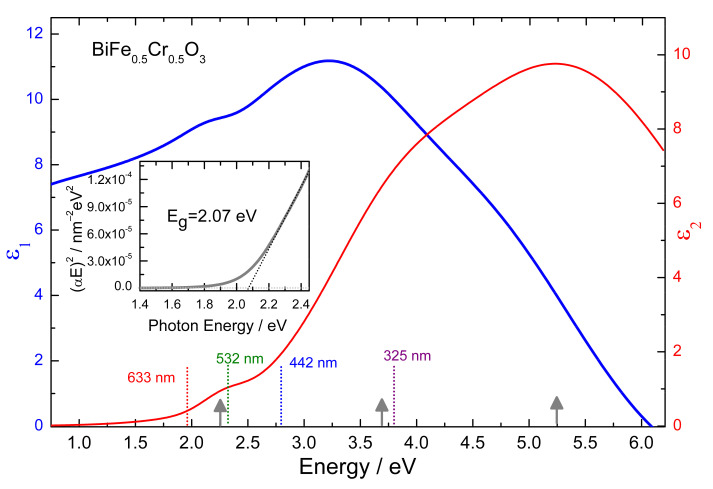
Real (ε_1_) and imaginary (ε_2_) part of BiFe_0.5_Cr_0.5_O_3_ dielectric function. The dotted vertical lines indicate the energy position of the laser lines used for Raman spectroscopy, while the vertical grey arrows show the energy position of the absorption peaks. The inset show the linear extrapolation of (αE)^2^ to zero absorption which give the value of the optical band gap.

The determined dielectric function of the BFCO is plotted in Figure 1 as a function of energy. The energy position of the Gaussian oscillators obtained from the fit at 2.27, 3.69, and 5.25 eV are marked in the same figure by arrows. It should be mentioned that also a 4th peak located at 9.6 eV, i.e., outside the measured range, had to be considered. In the same figure the energy position of the laser lines used as excitation for the Raman spectroscopy measurements are also indicated by dotted vertical lines.

**Figure 2:**
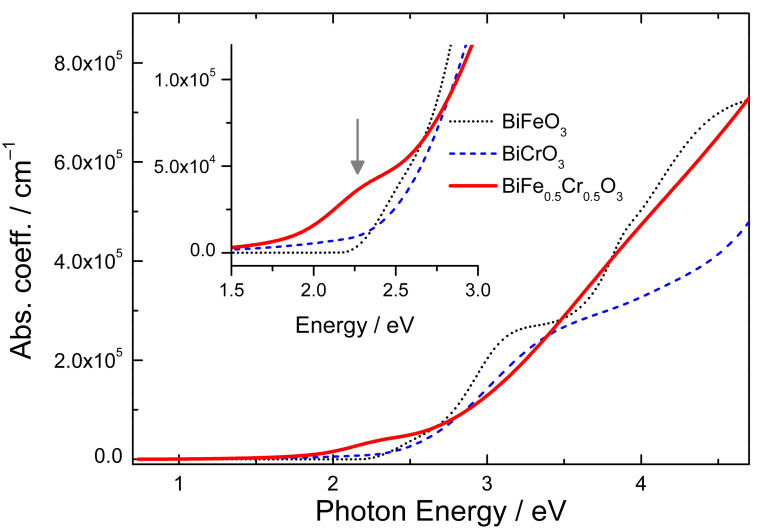
Absorption coefficient of BiFe_0.5_Cr_0.5_O_3_ in comparison with the absorption coefficients of BiFeO_3_ and BiCrO_3_. The inset show a zoom of the absorption onset region with the vertical arrow indicating the first absorption peak at ~2.27 eV.

From the real ε_1_ and imaginary ε_2_ part of the dielectric function, the complex refractive index *n* + *ik* is calculated. The absorption coefficient α can be derived from the extinction coefficient k as: α=4πk/λ. The absorption coefficient of BFCO is plotted in Figure 2 in comparison to the absorption coefficient of BiFeO_3_ and BiCrO_3_ films. It is very clear from the figure (especially from the zoom in the inset of Figure 2) that the BFCO films start to absorb at lower energies than its parent materials BiFeO_3_ and BiCrO_3_. The arrow at 2.27 eV indicates the position of the first absorption peak as determined from ellipsometry. These results corroborate that BFCO is promising material in the field of photovoltaic applications due to its extended absorption of the solar spectrum when compared with BiFeO_3_ or BiCrO_3_.

In order to gain a better look on the onset of the absorption, this region was analysed in detail. The optical gap of the BFCO films was determined by using the procedure for bandgap determination proposed by Tauc et al. [22] that was already successfully applied to BiFeO_3_ and BiCrO_3_ films [23,24,25]. 

Using the proportionality ${\left(\alpha E\right)}^{2}\sim E-E_{g}$ and extrapolating to zero absorption, a value of the energy gap E_g_ of 2.07 eV was obtained, as can be seen in the inset of Figure 1. This value is well below the values of the direct optical gaps of 2.8 eV or 2.95 eV reported for BiFeO_3_ [23,24] and BiCrO_3_ [25], respectively. The bandgap value obtained is, however, in good agreement with the values determined for Bi_2_FeCrO_6_ films which were found to be strongly influenced by the deposition conditions, cation ordering, domain size, or film thickness [9,10,12,13,26,27,28].

Considering the first peak determined in the imaginary part of the dielectric function, ε_2_, of BFCO at ~2.27 eV, its position is very close to the first absorption peaks observed in stoichiometrically similar Bi_2_FeCrO_6_ films [11] or in LaFe_0.5_Cr_0.5_O_3_ [29,30]. Its origin was attributed to a charge transfer excitation between Cr and Fe, being only present in the Fe/Cr mixed systems [11,29,30,31]. The lowest transition in Bi_2_FeCrO_6_ was also predicted to be between the Fe3d and Cr3d states using ab initio calculations in the pioneering work of Baettig et al. [1]. The appearance of this charge transfer band plays a crucial role in understanding the Raman spectra of BFCO that will be presented in the next section.

### 3.2. Raman Spectroscopy

**Figure 3:**
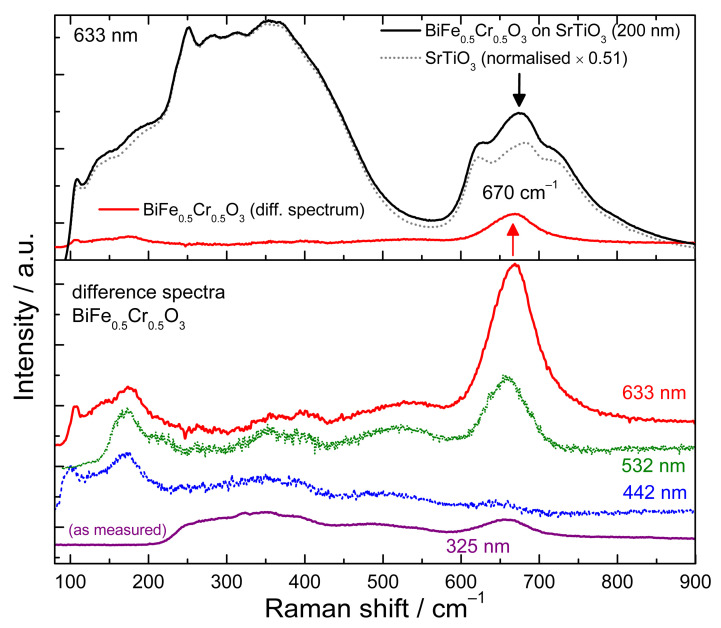
Raman spectra of a 200 nm BiFe_0.5_Cr_0.5_O_3_ thin film on SrTiO_3_ substrate and of the substrate normalised at the same intensity in the 200–400 cm^−1^ region, measured with an excitation of 633 nm. The corresponding film signal was calculated as a difference spectrum of the two (upper panel). The difference spectra of the thin film obtained with different excitations lines (633 nm, 532 nm, and 442 nm) and the as measured spectrum with the 325 nm excitation (lower panel).

The structure of BiFe_0.5_Cr_0.5_O_3_ is composed of alternating ABO_3_-AB’O_3_ perovskite cells (also called as double-perovskite structure A_2_BB’O_6_). Structural studies using XRD and neutron diffraction showed that at room temperature BiFe_0.5_Cr_0.5_O_3_ has a trigonal lattice of the space group *R3c* (No. 161) very similar to that of the BiFeO_3_ [32,33]. In the case of BiFeO_3,_ for this space group, group theory predicts 4A_1_ and 9E polar Raman modes [34,35,36]. The Raman spectrum of a 200 nm thick BiFe_0.5_Cr_0.5_O_3_ film deposited on the SrTiO_3_ substrate measured with an excitation of 633 nm is shown by the continuous line in the upper part of Figure 3. The Raman signal is dominated by the broad peaks of the substrate. In order to extract the BiFe_0.5_Cr_0.5_O_3_ signal, the substrate spectrum (dotted line in Figure 3) was measured by focusing the laser at a depth of about 30 µm below the film surface, and subsequently subtracted from the film/substrate spectrum. The difference spectrum is shown by the red line in the upper part of Figure 3. As can be seen, besides some weak peaks below 600 cm^−1^, there is a prominent peak at 670 cm^−1^. This peak was attributed in a previous Raman study of BiFe_0.5_Cr_0.5_O_3_ films to the SrTiO_3_ substrate due to its proximity to the substrate peak [18]. As can be seen in the lower panel of Figure 3, the 670 cm^−1^ peak is also present in the difference spectrum obtained with the 532 nm and nearly absent in the case of 442 nm excitation line, using the same procedure of subtraction for these excitations. Such a strong intensity dependence on the excitation wavelength is a clear indication of coupling phenomena.

Its origin will be discussed in the following part using also the ellipsometry data. The penetration depth d of light in a material can be calculated from the absorption coefficient α or from the extinction coefficient k as: d=1/α=λ/4πk. Considering the determined absorption coefficient (Figure 2) the penetration depths of the 633, 532, and 442 nm light are 750, 248, and 115 nm, respectively. This of course means that the laser light penetrates through the film and that also a substrate signal is expected when measuring 100–200 nm thick films. On the other hand, when measuring the Raman spectra using 325 nm excitation, which has a penetration depth of only 25 nm in BiFe_0.5_Cr_0.5_O_3_ the measured spectra correspond only to the film. As can be seen in Figure 3, the peak at 670 cm^−1^ is also present in the spectra recorded with the 325 nm excitation. This clearly indicates that the 670 cm^−1^ peak is intrinsic to BiFe_0.5_Cr_0.5_O_3_ and not to the substrate. Its strong intensity dependence on the excitation energy can be understood by the proximity of the energy of the excitation laser to the absorption peaks (see Figure 1). The 633 nm and 532 nm wavelengths are close to the first absorption peak, 325 nm is close to the second absorption peak, while 442 nm is far from such resonances and the Raman peak at 670 cm^−1^ is hardly observable for this last excitation.

It should be also mentioned that the Raman spectra for Bi_2_FeCrO_6_ previously published in the literature [18] show a very strong peak at ~830 cm^−1^ which was also found in the case of Cr doping of BiFeO_3_ or for BiFe_x_Cr_1−x_O_3_ powder, and was attributed to Cr-O vibration [19,37]. In our films, as it can be seen in Figure 3, we do not observe such a peak. However, such peak was found previously in different species of chromium oxides species [38,39,40,41]. The presence of this peak in the studies [18,19] may indicate that, in those cases, BiFe_x_Cr_1−x_O_3_ were not a pure phase and maybe contained species of chromium oxides.

**Figure 4:**
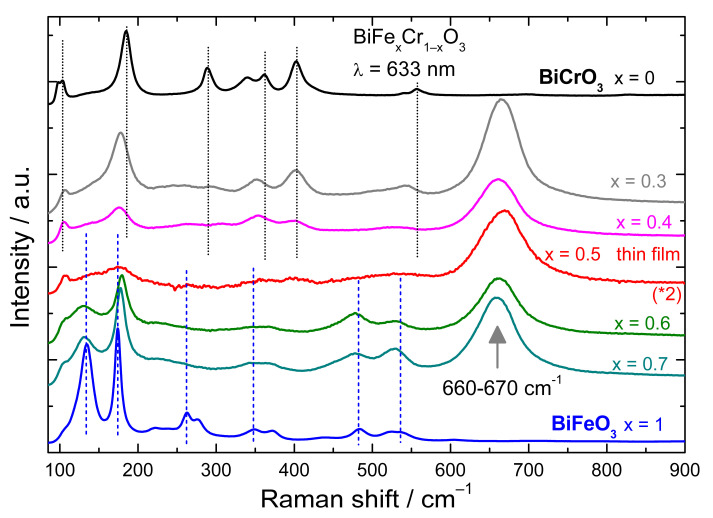
The difference spectrum of the BiFe_0.5_Cr_0.5_O_3_ thin film compared to the spectra of bulk BiFe_x_Cr_1−x_O_3_, and of the parent compounds BiCrO_3_ and BiFeO_3_ measured with 633 nm excitation. Note the strong peak at 660–670 cm^−1^ which is detected only for the samples with a Fe/Cr mixture.

In order to shed light on the origin of the 670 cm^−1^ peak, we measured Raman spectra of bulk BiFe_x_Cr_1−x_O_3_ (x = 0.3, 0.4, 0.6, 0.7) polycrystalline samples. The spectra measured with 633 nm excitation are shown in Figure 4 together with the difference spectra of the 200 nm BiFe_0.5_Cr_0.5_O_3_ film, and of the parent compounds BiFeO_3_ and BiCrO_3_. As can be seen, a peak at 660–670 cm^−1^ is present in all the spectra of Fe-Cr containing systems and absent for BiFeO_3_ and BiCrO_3_. On the other hand, no peak is measured between 800 and 900 cm^−1^, which indicates the absence of secondary chromium oxides phases in our thin films and bulk samples.

**Figure 5:**
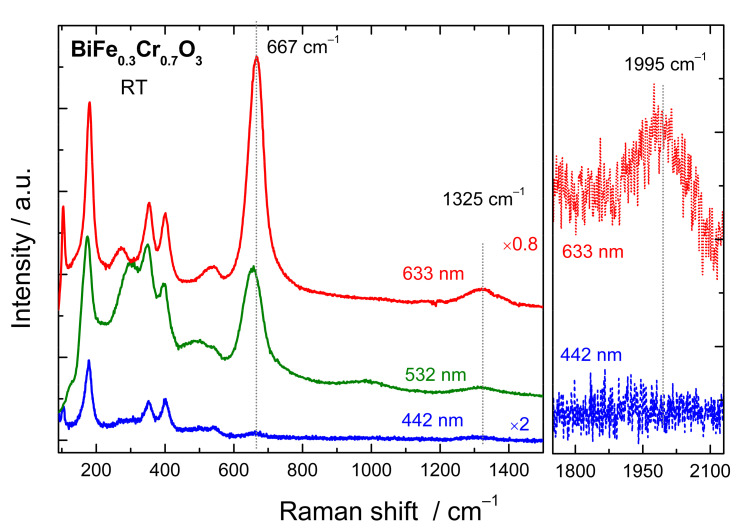
Comparison of the Raman spectra of bulk BiFe_0.3_Cr_0.7_O_3_ recorded with different excitation lines in the range of the first, second, and third order of the mode at ~667 cm^−1^.

For x = 0.7, 0.6, and 0.5 the Raman spectra are resembling more to those of BiFeO_3_, indicating the expected *R3c* space group. On the other hand, for x = 0.3 and 0.4 the spectra are more BiCrO_3_-like (*C2/c* space group) [25]. The relative intensity of the 660–670 cm^−1^ mode in respect to the polar mode (***A_1LO_****-**E_LO_***) at 172 cm^−1^ (BiFeO_3_-like) [36] or to the ***A_g_*** mode at 185 cm^−1^ (BiCrO_3_-like) [42] is strongest for the highest Fe-Cr mixture (x = 0.4, 0.5, and 0.6). This fact corroborated to the absence of the peak in the case of BiFeO_3_ and BiCrO_3_ indicate that the origin of the peak is related to the simultaneous presence of Fe and Cr in the structure. A very similar peak, at similar positions, has been measured also for other perovskite systems with Fe/Cr intermixing. For example, for HoFe_1_-_x_Cr_x_O_3_ a peak at 670 cm^−1^ was attributed to oxygen breathing mode (***A_g_***-like) and was found to be activated due to the charge transfer between Fe^3+^ and Cr^3+^ ions (mediated by O^2−^) playing a role in electron–phonon coupling [31]. As for our system, the mode was absent or having a very low intensity for the stoichiometry with only Fe or only Cr. Fe-Cr charge transfer effects activated a similar oxygen vibration mode related to the octahedron breathing in the case of LaFe_0.5_Cr_0.5_O_3_ [30] or LaFe_x_Cr_1−x_O_3_ systems [29,43] where the mode again vanished in the case of the parent compounds. All these aspects also indicate that in the case of BiFe_x_Cr_1−x_O_3_ the 670 cm^−1^ mode is an oxygen-related mode activated by the charge transfer between the Fe and Cr ion, mediated by the excitation light and is strongest when the energy of the incident photons is close to the first absorption peak at 2.27 eV (corresponding to charge transfer excitation between Cr and Fe). This was observed in case of our BiFe_0.5_Cr_0.5_O_3_ films, where the intensity of the 670 cm^−1^ peak was higher for 633 nm and 532 nm than for 442 nm excitation. Raman spectra for the BiFe_0.3_Cr_0.7_O_3_ polycrystalline sample measured with the same excitation lines are shown in Figure 5. Additionally, in this case the strongest peak at 667 cm^−1^ is measured when exciting with 633 nm and 532 nm. Moreover, the second order of this peak at 1325 cm^−1^ and even the third order at ~1995 cm^−1^ are observable for these two excitations (the third order peak for 532 nm is not shown due to the coincidence with some CCD artefacts), while for 442 nm even the first order is very low in intensity. A very similar behaviour was also observed for the other stoichiometries of the polycrystalline BiFe_x_Cr_1−x_O_3_ samples.

The multiple-order Raman scattering and the resonance effects were explained in the frame of the Franck–Condon type mechanism for rare-earth manganite systems [44,45,46]. The same Franck–Condon picture was also used to explain the Fe-Cr charge transfer which resonantly activates the oxygen breathing mode and its higher-orders. [31,43].

**Figure 6:**
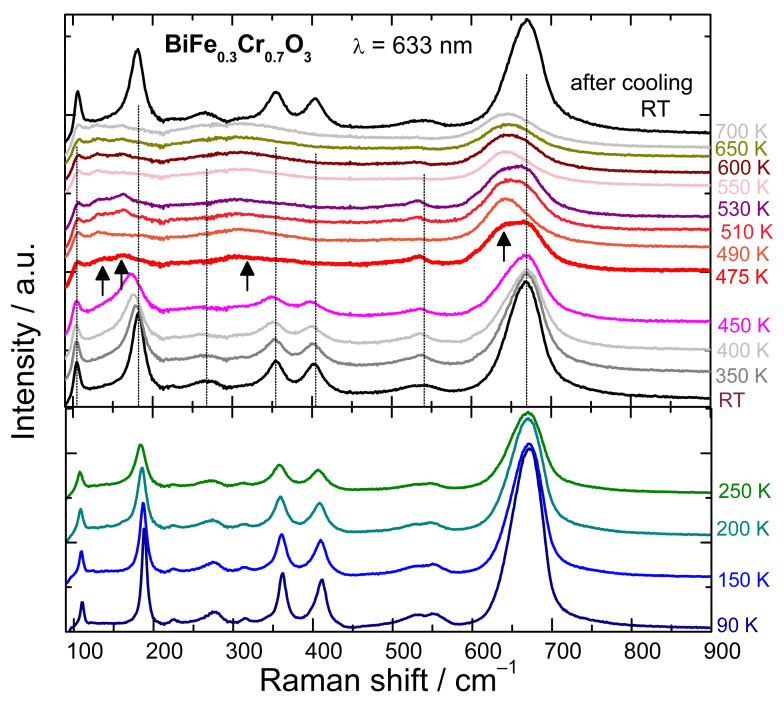
Temperature dependence (90–700 K) of the Raman spectra for bulk BiFe_0.3_Cr_0.7_O_3_ showing a reversible phase transition above 475 K.

The temperature dependent Raman spectra of BiFe_0.3_Cr_0.7_O_3_ polycrystalline sample in the 90–700 K temperature range are shown in Figure 6. With increasing the temperature up to 450 K the Raman peaks below 600 cm^−1^ are decreasing in intensity, shifting to lower wavenumbers and become broader, which is a characteristic thermal behaviour related to anharmonicity of the phonon vibrations. However, the position and the broadening of the mode at 670 cm^−1^ are much less affected by the temperature. This fact could be another indication that the activation of the mode is related to the Fe-Cr charge transfer. Starting with 475 K some new peaks are developing as indicated by the arrows in Figure 6, while peaks corresponding to the lower temperatures (vertical lines) are disappearing. Such a behaviour points to a structural phase transition. The new peaks which develop above 475 K are also present in the spectra measured at 700 K. After subsequent cooling at room temperature the same spectrum as before heating is obtained, indicating that the phase transition is reversible. In the case of BiCrO_3_, Raman spectroscopy revealed a reversible phase transition from *C2/c* monoclinic phase to a *pnma* orthorhombic phase above 400 K [25]. For BiFe_0.3_Cr_0.7_O_3_ the transition temperature increases. Syncrotron X-ray powder diffraction (SXRD) data indicates a *pbam* symmetry (PbZrO_3_-type) at room temperature while at 600 K the structure changes to *pnma* symmetry of GdFeO_3_-type (see Figure S2 in Supplementary Materials). The refined lattice parameters obtained from the Rietveld analysis method of the SXRD data by means of *RIETAN-2000* program [47], indicate that the phase transition is accompanied by a reduction in the volume of the unit cell by a factor of two.

## 4. Conclusions

In conclusion, in BiFe_0.5_Cr_0.5_O_3_ thin films and BiFe_x_Cr_1−x_O_3_ bulk appearance of a new Raman mode at ~670 cm^−1^ which is absent in the BiFeO_3_ and BiCrO_3_ parent compounds was evidenced by Raman spectroscopy. The mode was attributed to an oxygen (octahedral) breathing vibration which is mediated by the charge transfer between the Fe and Cr ions. This charge transfer corresponds to an optical absorption peak at ~2.27 eV for BiFe_0.5_Cr_0.5_O_3_ which lowers the bandgap of the material compared to the parent compound as was found by spectroscopic ellipsometry. On the other hand, Franck–Condon mechanism is manifested by a strong dependence of the intensity of the 670 cm^−1^ Raman peak on the excitation wavelength, and resonant activation of its higher scattering orders. For BiFe_0.3_Cr_0.7_O_3_ Raman spectroscopy revealed a reversible temperature induce structural phase transition.

## Data Availability

The data presented in this study are available on request from the corresponding author.

## Supplementary Materials

The following supporting information can be downloaded at: www.mdpi.com/article/10.3390/nano12091607/s1, Figure S1: X-Ray diffraction 2θ-ω scan comprising (001) and (002) peaks of STO substrate and 200 nm BFCO thin film (left panel). Topography scan (5 × 5 µm2) acquired by atomic force microscopy (contact mode) of the same BFCO thin film (right panel); Figure S2: High-temperature synchrotron powder X-ray diffraction measurements of the BiFe0.3Cr0.7O3 sample confirmed the existence of a reversible high-temperature structural phase transition from the PbZrO3-type structure (at room temperature) to the GdFeO3-type structure (at 600 K) [47,48].
