# Supplementary material for: Unexpected Phonon Behaviour in BiFexCr1−xO3, a Material System Different from Its BiFeO3 and BiCrO3 Parents
Source: Nanomaterials (Basel). 2022 May 9;12(9):1607. doi: 10.3390/nano12091607 (PMC9100047; doi:10.3390/nano12091607)
Supplement: Supplementary file 1 [file nanomaterials-12-01607-s001.zip › nanomaterials-1684589-supplementary.pdf]

nanomaterials

Supplementary Materials

Unexpected Phonon Behaviour in $BiFe_xCr_{1-x}O_3$, a Material System Different from Its $BiFeO_3$ and $BiCrO_3$ Parents

Cameliu Himcinschi [1,*], Felix Drechsler [1], David Sebastian Walch [2,3], Akash Bhatnagar [2,3], Alexei A. Belik [4] and Jens Kortus [1]

[1] Institute of Theoretical Physics, TU Bergakademie Freiberg, D-09596 Freiberg, Germany; felix.drechsler@student.tu-freiberg.de (F.D.); jens.kortus@physik.tu-freiberg.de (J.K.)
[2] Zentrum für Innovationskompetenz SiLi-nano, Martin-Luther-Universität Halle-Wittenberg, D-06120 Halle (Saale), Germany; david.knoche@physik.uni-halle.de (D.S.W.); akash.bhatnagar@physik.uni-halle.de (A.B.)
[3] Institut für Physik, Martin-Luther-Universität Halle-Wittenberg, D-06120 Halle (Saale), Germany
[4] International Center for Materials Nanoarchitectonics (WPI-MANA), National Institute for Materials Science (NIMS), Namiki 1-1, Ibaraki, Tsukuba 305-0044, Japan; alexei.belik@nims.go.jp
* Correspondence: himcinsc@physik.tu-freiberg.de

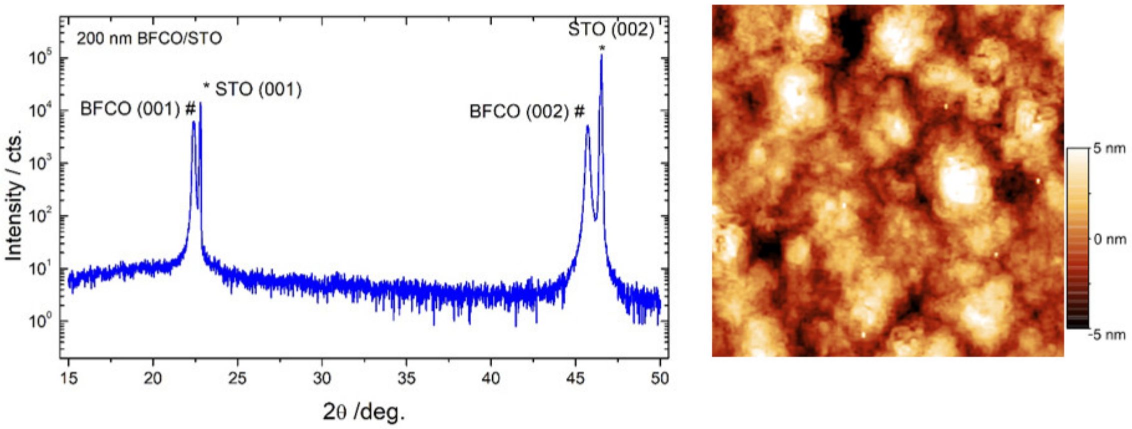

Figure S1. X-Ray diffraction 2θ-ω scan comprising (001) and (002) peaks of STO substrate and 200 nm BFCO thin film (left panel). Topography scan (5 × 5 μm²) acquired by atomic force microscopy (contact mode) of the same BFCO thin film (right panel).

The crystallinity and phase purity of the BFCO films were confirmed by X-ray analysis and a $2\theta - \omega$ scan around the (001) and (002) substrate peak (*) is presented in Figure S1 (left panel). The peaks of the film are marked by #. The growth under compressive epitaxial strain results in a smooth topography (AFM in Figure S1, right panel) with a root-mean-square roughness of ~ 1.98 nm.

Synchrotron X-ray Powder Diffraction (XRPD)

Experiments

Synchrotron X-ray powder diffraction (XRPD) data were measured between 300 K and 600 K on the beamline BL02B2 of SPring-8 [1]; the intensity data were collected between 2.08° and 78.22° at 0.006° intervals in 2θ and the incident beam was monochromatized at λ = 0.42014 Å. The samples were put into Lindemann glass capillaries (inner diameter: 0.1 mm), which were rotated during measurements. Synchrotron XRPD data were analyzed by the Rietveld method using the *RIETAN-2000* program [2].

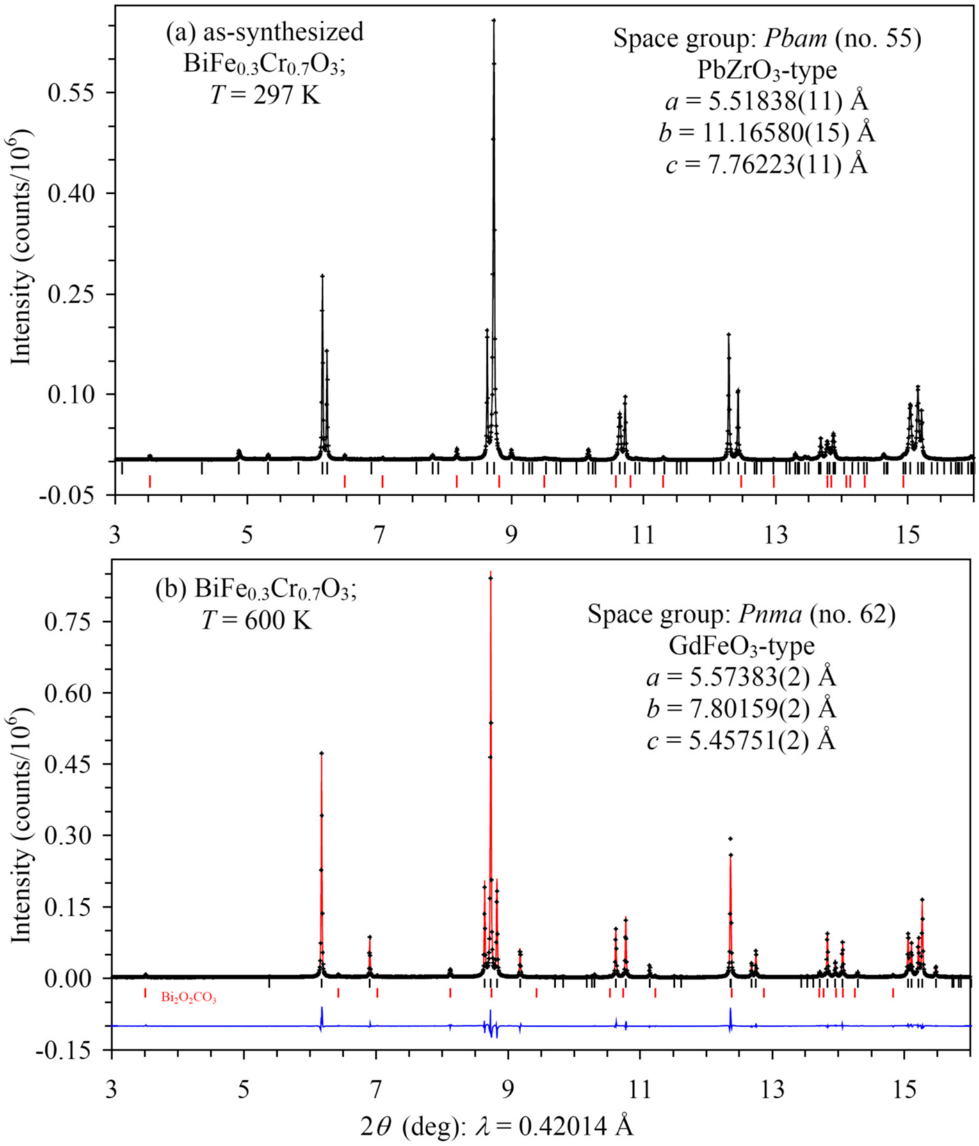

Figure S2. High-temperature synchrotron powder X-ray diffraction measurements of the $BiFe_{0.3}Cr_{0.7}O_3$ sample confirmed the existence of a reversible high-temperature structural phase transition from the $PbZrO_3$-type structure (at room temperature) to the $GdFeO_3$-type structure (at 600 K).

Experimental synchrotron XRPD data are shown by black crosses, and the calculated curve is given by line. Blue line shows a difference curve between the experimental and calculated data at 600 K. The tick marks show possible Bragg reflection positions for the main $BiFe_{0.3}Cr_{0.7}O_3$ phase (the first black row) and $Bi_2O_2CO_3$ impurity (the second red row). The refined lattice parameters in the corresponding models are listed on the figure.

References

1. Kawaguchi, S.; Takemoto, M.; Osaka, K.; Nishibori, E.; Moriyoshi, C.; Kubota, Y.; Kuroiwa, Y.; Sugimoto, K. High-throughput powder diffraction measurement system consisting of multiple MYTHEN detectors at beamline BL02B2 of SPring-8. *Rev. Sci. Instrum.* **2017**, *88*, 085111.
2. Izumi, F.; Ikeda, T. A Rietveld-analysis program RIETAN-98 and its applications to zeolites. *Mater. Sci. Forum* **2000**, *321–324*, 198–205.
